# The Legacy of the Pandemic Preparedness Regime: An Integrative Review

**DOI:** 10.3389/ijph.2022.1604961

**Published:** 2022-12-05

**Authors:** Mathilde S. Bourrier, Michael J. Deml

**Affiliations:** ^1^ Department of Sociology, Institute of Sociological Research, University of Geneva, Geneva, Switzerland; ^2^ Department of Quality and Health Technology, SHARE Center, Faculty of Health Sciences, University of Stavanger, Stavanger, Norway; ^3^ Division of Social and Behavioural Sciences, School of Public Health & Family Medicine, University of Cape Town, Cape Town, South Africa

**Keywords:** COVID-19, pandemic, H1N1, whole-of-society, preparedness

## Abstract

**Objectives:** The global response to COVID-19 inherited a long history of preparedness features pertaining to various threats, including bioterrorism, (re)-emerging infectious diseases, and pandemics. We describe the evolution of pandemic preparedness frameworks, before and after the COVID-19 pandemic.

**Methods:** We conducted an integrative literature review of publicly available documents, including grey and scientific literature, on pandemic preparedness frameworks. We relied on social science literature as a main source and used search keywords: pandemic preparedness, H1N1, COVID-19, “whole-of-society”/“whole-of-community.”

**Results:** The H1N1 pandemic (2009–2010) tested pandemic preparedness frameworks. Lessons-learned reports concluded that the global H1N1 response were too strong and unnecessarily alarming. Such critiques, pandemic fatigue, and budgetary cuts post-2008 explain lack of preparedness for COVID-19. Critiques culminated in a shift towards a “whole-of-society” approach to health crises, although its uptake has not been ideal.

**Conclusion:** Traditional preparedness regime limits arose again during the COVID-19 pandemic. The “whole-of-society” approach was not fully deployed in COVID-19 responses. A “whole-of-organizations” approach could be designed, ensuring that countries consider local organizations’ potential to partake in containing infectious disease and counter undesirable side-effects of non-pharmaceutical measures.

## Introduction

More than two and a half years into the COVID-19 pandemic, it’s time for some retrospective reflection on where we were standing before it all began. Pandemic preparedness has been a major topic in global and public health circles for decades. The United Nations (UN) and the World Health Organization (WHO) define preparedness as “the ability (knowledge, capacities, and organizational systems) of governments, professional response organizations, communities, and individuals to anticipate, detect and respondeffectively to, and recover from, the impact of likely, imminent or current health emergencies, hazards, events or conditions. It means putting in place mechanisms that will allow national authorities, multilateral organizations and relief organizations to be aware of risks and deploy staff and resources quickly once a crisis strikes” (1, p. 16). Before COVID-19, the possibility that a devastating pandemic might sweep the world was not at all unthinkable. On the contrary, numerous public health officials, global health experts, and public health scholars have recurrently warned that it was not an “if” but rather a “when.”

Pandemics are increasing in frequency due to increasing deforestation, climate change, urbanization, globalization, and possibly dangerous interconnections between animal and human habitats [2]. In the midst of the Ebola pandemic in West Africa, then WHO Director General Margaret Chan famously declared, “The World is ill-prepared to respond to any severe, sustained, and threatening public health emergency” at the 64th session of WHO Assembly (WHO, 2015). This statement echoes an almost identical one made 5 years earlier in 2010 after the H1N1 pandemic by the International Health Regulations (IHR) Review Committee, which had been commissioned to assess the response to the 2009–2010 H1N1 pandemic: “The world is ill-prepared to respond to a severe influenza pandemic or to any similarly global, sustained and threatening public-health emergency” [3].

After going through the main established pillars of pandemic preparedness before 2020, we will focus on how the doctrine was received at the time. We will go back to main debates that such a vision on pandemic preparedness had raised. Our goal is to see how that state of mind can explain what happened in the first few months of 2020. Our objective is to ask the following research questions: How has social science literature discussed the pandemic preparedness regime prior to the COVID-19 pandemic? What kind of frameworks were available at the beginning of the COVID-19 pandemic?

## Methods

We conducted an integrative literature review [4, 5] of publicly available documents, including grey and scientific literature, to present the main frameworks guiding pandemic preparedness at the onset of COVID-19. We purposively selected social sciences literature as our main, but not exclusive, source of documentation, using the following keywords in Google Scholar: COVID-19, pandemic, H1N1, whole-of-society, preparedness. We have opted to undertake a integrative review and not an exhaustive one. Preparedness is a social construct that is often discussed in social science and social anthropological literature. However, to our knowledge, there are no large-scale empirical analyses about pandemic preparedness at the level of organizations, which renders systematic reviews difficult.

We sought to bring back to the forefront the debates that had been at the core of the preparedness regime pre-COVID-19, to not forget where we were coming from. Our text is also informed not only by the current contribution both authors make to Norwegian-based project PAN-FIGHT [6], but also by earlier work on vaccine hesitancy [7–10] and on the global management of pandemic responses prior to COVID-19, namely H1N1 and Ebola [11]. PAN-FIGHT is a 5-country research consortium that compares the risk communication strategies and public health mitigation measures implemented in Germany, Norway, Sweden, Switzerland, and the United Kingdom in 2020 in response to the COVID-19 pandemic [12].

## Results

In the following sections, we repatriate debates that occurred during the first 2 decades of the 21st century and focus particularly on those following the H1N1 pandemic (2009–2010). Such debates have been extensively researched and documented in Bourrier et al (2019) [11]. We describe how the H1N1 pandemic had already put the preparedness apparatus to the test, with mixed results and critiques emerging from how it was handled. We then discuss how a new approach developed following these critiques. This “whole-of-society” approach was proposed at the international level by WHO [13], and the US Federal Emergency Management Agency (FEMA) used the term “Whole Community” [14]. Experts in the field of preparedness acknowledged that top-down mitigation measures would not be sufficient to build an adequately powerful response capable of saving lives and sustaining communities during pandemics. With such an approach, full ownership of and participation in developing protective measures would need to come from diverse segments of the society, in a bottom-up fashion. As a FEMA document defines the concept, “Whole Community is a means by which residents, emergency management practitioners, organizational and community leaders, and government officials can collectively understand and assess the needs of their respective communities and determine the best ways to organize and strengthen their assets, capacities, and interests” (14, p. 3). We then examine how the “whole-of-society” approach fared in the context of COVID-19. Signaling some limitations to this approach, we conclude this analytical review by suggesting that a “whole-of-organizations” approach, as a complement to the “whole-of-society” approach, could be designed, ensuring that countries consider the potential of local institutions and organizations to join forces in containing the spreading of the disease as well as counter social and economic side-effects [15] of non-pharmaceutical measures (furloughs, unemployment, increased stress, dropouts at school, depression, loneliness, isolation).

### The Preparedness Regime Before COVID-19

The 9/11 attacks in New York provided a strong impetus for the development of emergency preparedness as a dominant way of conceiving domestic security and risk and of managing uncertainty in the United States [16, 17] and elsewhere. The paradigm includes emerging and re-emerging infectious diseases, like Ebola, Zika, SARS, MERS and influenza viruses like H1N1, combined with the threat of the Avian Flu H5N1. The paradigm dominated the first 2 decades of the new century. The emergence and resurgence of these viruses triggered a whole range of instruments, which aimed at linking governments, national and international health authorities (CDC and WHO), big pharma, and surveillance systems. Influenza attracts mass public health efforts and has been the subject of a global surveillance network, the Global Influenza Surveillance and Response System (GISRS), since its inception in 1951 [18, 19]. The revision of the International Health Regulations (2005) was an attempt to heighten the level of surveillance and compliance of Member States facing epidemics of pandemic potential, especially respiratory viruses. Since the turn of the 21st century, WHO has been pivotal in identifying and combating the spread of at least six major epidemics it defines as pandemics. These include [1]: SARS in 2002–2003 [2]; the Avian Flu (H5N1) in 2004 [3]; the Swine Flu (AH1N1) from 2009 to 2010 [4]; MERS in 2012 [5]; Ebola from 2014 to 2016; and [6] COVID-19 pandemic. Pieri (2021) borrows from the WHO definition to define a pandemic as “an outbreak of a new infectious disease that spreads across countries and may have the potential to spread worldwide” (20, p. 7).

During this time period, public health experts from all over the world launched a vast operation to draft pandemic plans, largely at the instigation of the WHO’s 2004 “experts’ consultation on priority public health interventions before and during an influenza pandemic” [21]. By doing this, WHO recommended that all countries develop preparedness plans for a potential pandemic and provided support to countries through the development of an arsenal of “evidence-based” guidance. These preparedness plans were primarily based upon potential responses to, and lessons learnt from, influenza epidemics. The “mother” of all pandemics remained the Great Influenza pandemic of 1918 which killed an estimated 50 to 100 million people throughout the world [22–24].

Diverse organizations operating in different sectors (mainly health institutions, public utilities, transportation companies, national, regional and local authorities but also banks, among others) have subsequently dedicated a significant amount of time year after year to implement this fourfold operation [1]: to sensitize their own organizations [2], to set up contingency plans in case of a pandemic [3], to anticipate the needs for masks, hospital beds, drugs, vaccines, PPE and other protective materials, and [4] to envision potential impacts of expected absenteeism due to the flu on economic and collective life [11, 25–28].

This pandemic preparedness regime has led to various implementations of pandemic plans, scenario planning exercises, and national and international strategies in anticipation of potential pandemics. A full doctrine has been developed and shared between international organizations (WHO, World Bank), large philanthropic organizations (Bill and Melinda Gates Foundation, Clinton Foundation), Gavi, the Vaccine Alliance, the Wellcome Trust and other global health powerhouses, like the Centers for Disease Control and Prevention (CDC) in the United States, to increase the level of preparation of states and their respective governments. Despite such strong encouragement to engage in preparedness efforts, it has not been met with full enthusiasm everywhere. Countries responded diversely, and this was the case at the regional, local, and organizational level [1]. It is interesting to note that the Global Security Index in 2019 ranked the USA and the UK as the top 2 countries in terms of preparedness; yet, both countries recorded some of the worst COVID-19 excess mortality rates [29].

At the international level, a range of documents related to influenza pandemic preparedness had been published by WHO before 2020 [1]: “Pandemic influenza risk management: WHO interim guidance” [2, 30] “Pandemic influenza risk management: A WHO guide to inform & harmonize national & international pandemic preparedness and response” [3, 31] “A checklist for pandemic influenza risk and impact management: Building capacity for pandemic response” [4, 32] “Essential steps for developing or updating a national pandemic influenza preparedness plan” [33]; and [5] “Non-pharmaceutical public health measures for mitigating the risk and impact of epidemic and pandemic influenza” [34]. The drafting and continuous updating of these documents attest to the importance of influenza preparedness to the WHO. In 2019, prior to COVID-19, WHO had just released its global influenza strategy 2019–2030 [35].

At the national level and as an example, since 2000, all five countries of the PAN-FIGHT sample (Switzerland, UK, Germany, Sweden and Norway) have developed, revised, and updated their overarching national plans preparing for, responding to, and recovering from potential pandemics [36–40]. All five countries had been affected by the H1N1 epidemic in 2009–2010 and attempted to implement vaccination campaigns with some variations in their strategies [41]. All plans have been updated following the H1N1 pandemic (2009–2010). In addition to pandemic planning, some countries developed scenario planning exercises. Pandemic simulations and modelling are increasingly common features of the preparedness apparatus that are used by policymakers and responders “to inform rapid decision-making in near to real-time, both before and during a pandemic event” (20, p. 156). The UK for example conducted several exercises (Exercise Cygnet in 2016, Exercise Cygnus in 2016, and Exercise Iris in 2018) to test preparedness. The reports from these exercises concluded that there were gaps in preparedness for pandemics. Germany also simulated an influenza pandemic exercise in 2007 called LÜKEX 07, to train cross-state and cross-department crisis management. In 2017 during the time of the G20 meeting, Germany ran a health emergency simulation exercise with WHO and World Bank representatives to prepare for potential future pandemics. Meanwhile, Switzerland referred to SARS and H1N1 outbreaks in updating its legislation guiding its response to epidemics [12].

### H1N1 Puts the Preparedness Regime to the Test

Overall, the H1N1 pandemic put this preparedness regime to the test, with mixed results. To a large extent, the above-mentioned pandemic plans proved, at least according to retrospective declarations [42], to be helpful logistically-speaking. They were used as a coordination mechanism but were ultimately set aside because the plans compelled action based on a severe crisis, whereas the threat proved to be less severe than anticipated [28]. Various investment logics came into play and concerned the resources that needed to be mobilized, resources that were not only financial in nature, but also organizational, communicational, and cognitive. The establishment of pandemic plans, along with contingency and business continuity plans within administrations, hospitals, schools, public transportation, the private sector, airports and places with high concentrations of people, fell on the responsibility of hundreds of professionals (in various capacities, from the upper ranks to the lower ranks of hierarchies) in anticipation of a potential pandemic, including small and medium-sized organizations [43].

The rolling out of the thought-out plans for one of the most severe pandemics as well as the difficulty of leaving behind a worst-case-scenario logic characterized the response to the H1N1 pandemic [43]. Generally, it was noted that plans were designed for a severe pandemic and lacked flexibility [28]. Many of the responders throughout the world, particularly those who worked in national public health services, attributed this escalation to the strong injunctions provided by the international echelon represented by WHO. WHO top leaders were accused of having a “cry wolf attitude” [44]. More radically, WHO officials and notably members of the Emergency Committee advising WHO on the response have been accused of being subject to undue pressure by pharmaceutical industries in order to favor vaccination against H1N1 [45].

The variation in H1N1 pandemic responses was striking across European countries, even between countries with very similar profiles [46]. Despite strong international impetus, similar threats, and comparable resources invested at the country level, European countries offered a picture of contrasts. This is especially clear when considering vaccination campaigns. Some countries, such as Switzerland and France, aimed for extensive vaccination, while others, such as Denmark, aimed for more targeted vaccination. This was especially the case in early stages, when there were not yet enough vaccines to consider mass vaccination [41]. In addition, these campaigns were met with very different degrees of social acceptance [41, 46]. To summarize, the response to H1N1 was mainly geared towards the production of a vaccine. However, the time it took to produce it, and the difficulties encountered in convincing populations to get vaccinated, provoked social controversies, which consistently made headlines in the press [47].

Experience with the H1N1 pandemic provided international organizations, like the WHO and Member States, with a benchmarking exercise, from which to measure their level of readiness [48]. H1N1 was not as virulent as COVID-19, although at the beginning some elements, such as young adults and children displaying higher mortality rate in rare cases [49], triggered a large-scale response. Fortunately, H1N1 did not prove to be as lethal as feared early on [50]. After the fact, numerous lessons-learned reports concluded that the global response had been too strong and unnecessarily alarming [45, 51–56]. Resulting criticisms and pandemic fatigue plagued preparedness thinking after H1N1([27, 28, 57]) and this period was characterized by budgetary austerity following the 2008 recession. These factors contribute to understanding the lack of preparedness for COVID-19 [58].

After the H1N1 crisis, and in line with the observations made in the retrospective feedback reports, Member States adopted an ambivalent position towards the preparedness framework. This ambivalence allowed them to free themselves of the oversight of the WHO in matters of risk assessment, while at the same time recognizing WHO’s central role. A change in vocabulary appeared: instead of “plans,” “checklists” and “steps” became the preferred terms. At the instigation of WHO, some countries—like Sweden—have also moved away from specific pandemic preparedness plans to incorporate a wider range of emergencies, and have built more generic plans, that could be of use in several types of emergencies and not only during health emergencies. The “all-hazards” doctrine, which had already existed prior to H1N1, began to slowly regain ground in frameworks for thinking about pandemic preparedness [59].

We hypothesize that this past history with H1N1, only 10 years prior to the COVID-19 pandemic, has played an influential role in the early days of the global response in 2020. In their responses to both H1N1 and COVID-19, European countries diverged greatly in their approaches [60, 61]. It is also of importance to recall that H1N1 vaccination campaigns were met with social controversies. In the aftermath of H1N1, the conventional wisdom captured in the corridors of different organizations [42] was that international and national authorities triggered excessive responses (with some exceptions, such as the United States) and failed to propose more tailored and adequate pandemic responses.

### Looking Beyond the Traditional Preparedness Regime

Anthropologist of science and medicine, Andrew Lakoff signaled at the end of his book Unprepared [62] that the preparedness project in itself is doomed to fail firstly because of “two conundrums, one temporal and one spatial” (26: 167). The temporal conundrum points to the difficulty of accounting for potentially never-ending emergencies of a global nature. At the same time, the regime itself offers a framework built around a circumscribed “response” whose spatial print is nonetheless complex to apprehend. Notably because it includes local, regional, national, and often international dimensions. Early detection, gathering expertise, and organizing responses to epidemics entails different scale and scope. Secondly, the preparedness regime masks fundamental health inequalities throughout the world.

Managing responses from centrally located institutions runs the risk of favoring standardized approaches, narrowing down options, and overlooking local contexts. These underlying structural inequalities imply that: 1) surprises will happen; 2) the capacity to play by scenario-planning is questionable; 3) no matter how well the pandemic plan is drafted, if vital infrastructures are weak, the response cannot be strong. Similarly, Forster [45] argues how “universalistic and one-size fits all responses” could be damaging and misguiding.

However, as explained by Bastide [17], the preparedness regime had already tried to adapt in response to these flaws through the development of the “whole-of-society approach.” The approach responded to the idea that large-scale disasters were imminent and that in such a case, it was likely that the government resources and capacities would be overstretched and insufficient. To overcome this overflow, the whole community approach is designed to diffuse preparedness capabilities throughout communities, turning “individuals [into] subjects of preparedness” (5:34). Central entities within the model include civil society, businesses, and governments. Strengthening communities, local schools, using social media, and developing educational programs was part of the new philosophy. Facing threats and catastrophes would require individuals to be resilient and capable of empowering themselves for their communities. The key is to “[embed] preparedness in the course of ordinary social process and practices in order to build resilience ‘within’ communities and within individuals” (5: 34).

In 2019, already prior to COVID-19, the WHO issued a document to further clarify this whole-of-society approach: “A whole-of-society approach aims to extend the whole-of-government approach by placing additional emphasis on the roles of the private sector, civil society and political decision-makers, such as parliamentarians. (…). A whole-of-society approach goes beyond institutions; it influences and mobilizes local and global culture and media, rural and urban communities and all relevant policy sectors, such as the education system, the transport sector, the environment and even urban design” [63]. Despite this clarification months before the beginning of the COVID-19 pandemic, the Global Preparedness Monitoring Board (2019) noted that “Efforts on national and local preparedness planning too often lack an effective “whole-of-government” and “whole-of-society” approach” (p. 24). A notable exception comes from the case of Taiwan, whose “whole-of-society” epidemic preparedness model was analyzed both before [64] and during [65] the COVID-19 response, with both analyses demonstrating how the country had established multi-sectoral, government-society collaboration mechanisms as an integral part of its preparedness strategy.

### The Preparedness Regime, the Whole-Of-Society Approach, and COVID-19

What happened when COVID-19 struck? What has been the fate of the “whole-of-society” approach? During the early weeks of the response, throughout the world and notably within high-income countries, governments did not seem to be ready for such a massive epidemic. For example, let’s consider the countries involved in the PAN-FIGHT study: Germany, Norway, Sweden, Switzerland, and the UK. Despite current health expenditures (% of GDP) 11.43% (2018) in Germany, 10.05% (2018) in Norway, 10.90% (2018) in Sweden, 11.88% (2018) in Switzerland, and 10.00% (2018) in the UK—mong the highest expenditures in the world -, all five countries experienced ruthless surprises and struggled with their COVID-19 responses [12]. In early 2020, masks, PPE, and functioning lines of coordination across countries, regions, and sectors were lacking.

Many organizations, both from the public and private sectors, did not activate their contingency plans, and many were not updated. Cappelli [66], for example, writes about this surprise that even though contingency plans had been worked out and prepared a decade earlier following experience with H1N1, in many cases, they have not been maintained properly. Whereas prior to the 2009 H1N1 pandemic, when business contingency planning was a priority, it seemed to have vanished in the COVID-19 response due to the mild case that H1N1 had represented (66: xv). Considered to be vastly exaggerated and too scaled up during the H1N1 pandemic, the preparedness impetus faded away after 2011, leaving many organizations exposed. There are, however, notable exceptions in banking, utilities, and healthcare institutions where contingency planning remained important.

This leads to the legitimate comment Caduff, author of The Pandemic Perhaps [67], makes, “What this pandemic shows is a lack of preparedness. This will come as a surprise, given the billions of dollars, euros, and pounds that were spent over the last 15 years on pandemic preparedness, including experience with past epidemics and pandemics such as Ebola and swine flu. How can it be that hospitals ran out of N95 in week one? [...] It will be important to understand why key preparedness concepts were sidelined in the pandemic, despite the attention that preparedness received and the substantial resources it consumed for over a decade” ([68], p. 480).

Few articles published since 2020 make explicit references to the “whole-of-society” approach [69, 70]. WHO officials and authorities throughout the world have mentioned the notion during the early months of the crisis [71] and some rare policy reports have also surfaced [72]. For example, in October 2020, at the United Nations General Assembly event on ‘Sustainable preparedness for health security and resilience: Adopting a whole-of-society approach and breaking the “panic-then-forget” cycle,’ WHO Director-General Tedros Adhanom Ghebreyesus stated, “Over the years we have had many reports, reviews and recommendations all saying the same thing: the world is not prepared for a pandemic. COVID-19 has laid bare the truth: when the time came, the world was still not ready” [73]. He proposed the solution of investing in preparedness, with an emphasis on an all-of-government and all-of-society approach. The approach might have been helpful in making sense of the situation experienced by numerous communities across the globe.

Health infrastructures were of course key, but the undisputable manifestation of the necessity to reach out to larger sections of a society than solely to health authorities was also integral to the response. Numerous associations and organizations, including NGOs, local stores, schools, and closed theaters, among others, have rallied in attempts to support local communities. In the medical sector, retiring health professionals came back to help, medical students took shifts at hospitals, some non-medical staff were repositioned to help in COVID-19 wards. Hospitals throughout the world had to tap into volunteering resources. We have collectively witnessed mass food distribution efforts [74], people handcrafting masks and other PPE [75], provision of shopping help to vulnerable or elderly persons, and a multitude of initiatives from NGOs, civil society, businesses in transport, travel, trade, finance, security and other sectors. However, what could really be interpreted as a whole-of-society approach being organically and spontaneously put into practice has yet to be fully understood [70]. A comparative report published by OECD in 2022 [76] acknowledges: “measures deployed to coordinate a whole-of-society response, such as mechanisms for cooperation across different levels of government or for engaging stakeholders in key decision-making processes, have not been sufficiently analyzed.”

## Discussion

This puzzling lack of pandemic preparedness for COVID-19 has yet to be fully explained. As mentioned above, pandemic fatigue after H1N1 can probably account for some of it. The harsh controversies that plagued public health authorities probably impeded their capacities to adequately engage different societal segments to stay alert and continue to invest in preparedness. Even if national plans are up to date, if the preparedness efforts do not trickle down to the regional, community, or organizational levels, their implementation remains abstract. After the H1N1 pandemic, the logic of overly prescriptive pandemic plans has been criticized. Member States relayed their desire for more freedom in terms of risk assessment to the WHO. WHO and other major public health institutions had also subsequently advocated for a more cross-sectoral approach. Various stakeholders expressed their interest in shifting pandemic preparedness plans into simpler checklists of must-haves. However, massive shortages of masks, PPE, and basic painkillers–such as paracetamol–across Europe notably, at the beginning of the pandemic, have demonstrated how such a shift has proven problematic.

Furthermore, 10 years is a long time for contemporary organizations. The efforts made during the first decade of this century and during H1N1 may not have been sustained in the second decade. Professionals move from one position to another, from one organization to another, and institutional memory is not always transmitted adequately. It remains to be seen how organizations can in general foster, support, and nurture their memory capacities. The idea is not to push for old recipes which could encourage a conservatism bias, when of course sometimes situations are radically different and call for new frameworks. It is as important to favor continuity in crucial investments (in ideas, materials, and frameworks alike), as it is to keep questioning these very investments. Lessons learned from past pandemics cannot only be kept in public reports and documents, scientific journal articles, and books [20]. They need to be continuously revisited to orient actions and decisions [77]. Some frameworks and lessons often fail to be incorporated into current logics and practices. They are set aside, as if completely forgotten, because they might prove to be uncomfortable, or question too many taken-for-granted premises, which are difficult to shake [78]. It is probable that the “whole-of-society” approach, although interesting conceptually and far-reaching in ambition, was not easily accommodated by governmental ministries and regional bodies of all kinds when it emerged. COVID-19 offers a unique opportunity to articulate and substantiate the notion.

In addition, what could be called a “whole-of-organizations” approach during COVID-19 also needs to be properly and systematically documented and analyzed. What really happened in a myriad of organizations, sectors, companies, small and big, private and public, during the different phases of the pandemic is only starting to be documented [79]. Reservoirs of ideas, alternative work practices, alternative coordination mechanisms have all proven central to the response. A top-down, centralized, bureaucratic response was not suited to leverage the many challenges that densely populated cities in particular had to face. That was expected. However, the probably better suited “whole-of-society” approach has not seemed to fare better either. Pieri (2021) has advocated for participatory crisis governance as a complement: “Citizens must be engaged along with other stakeholders in an all-of-society approach to planning and execution of mitigation. The mobilization of social science expertise alongside the expertise already dominant in pandemic planning is also key to facilitating public engagement” (20, p. 181–182). Along these lines, a new preparedness philosophy might emerge from the COVID-19 pandemic, maybe revolving around a “whole-of-organizations” approach, aiming at fostering organizational capacities in responding to cross-sectoral crises [80].
